# Ferulic Acid Supplementation Increases Lifespan and Stress Resistance via Insulin/IGF-1 Signaling Pathway in *C. elegans*

**DOI:** 10.3390/ijms22084279

**Published:** 2021-04-20

**Authors:** Hui Li, Xiaoxuan Yu, Fanwei Meng, Zhenyu Zhao, Shuwen Guan, Liping Wang

**Affiliations:** 1 Key Laboratory for Molecular Enzymology and Engineering, The Ministry of Education, Jilin University, Changchun 130012, China; lihui18@mails.jlu.edu.cn (H.L.); guanshuwen@jlu.edu.cn (S.G.); 2 School of Life Sciences, Jilin University, Changchun 130012, China; yxx18@mails.jlu.edu.cn (X.Y.); jlmfw@126.com (F.M.); zhenyu18@mails.jlu.edu.cn (Z.Z.); 3 Engineering Laboratory for AIDS Vaccine, Jilin University, Changchun 130012, China

**Keywords:** ferulic acid, lifespan, stress resistant, insulin/IGF-1, *Caenorhabditis elegans*

## Abstract

Ferulic acid (FA) is a naturally-occurring well-known potent antioxidant and free radical scavenger. FA supplementation is an effective strategy to delay aging, but the underlying mechanism remains unknown. In the present study, we examined the effects of FA on lifespan extension and its mechanism of FA in *Caenorhabditis elegans* (*C. elegans*). Results suggested that FA increased the lifespan of *C. elegans*, rather than altering the growth of *E. coli* OP50. Meanwhile, FA promoted the healthspan of *C. elegans* by improving locomotion and reducing fat accumulation and polyQ aggregation. FA increased the resistance to heat and oxidative stress through reducing ROS. The upregulating of the expression of the *hlh-30*, *skn-1*, and *hsf-1* were involved in the FA-mediated lifespan extension. Furthermore, FA treatment had no impact on the lifespan of *daf-2*, *hlh-30*, *skn-1*, and *hsf-1* mutants, confirming that insulin/IGF-1 signaling pathway and multiple longevity mechanisms were associated with the longevity mechanism of FA. We further found that mitochondrial signaling pathway was modulation involved in FA-mediated lifespan extension. With the results from RNA-seq results and mutants lifespan assay. These findings contribute to our knowledge of the lifespan extension and underlying mechanism of action of FA in *C. elegans*.

## 1. Introduction

Aging is accompanied by constant decline in physiology, an increase in mortality, and a decline in fecundity at later adult ages [1]. Aging can be delayed by lifestyle modulation involving smoking cessation, improving diet, reducing alcohol consumption, getting enough sleep, reducing stress, or consuming food supplements such as antioxidants to increase health benefits [2]. Many epidemiological studies have shown that natural bioactive products delay aging and reduce the risk of aging-related diseases [3]. Although aging is inevitable, the process of aging can be modulated and regulated to a significant extent. Several natural bioactive products and plant extracts exhibit lifespan extension abilities, including caffeic acid [4], curcumin [5], blueberry extracts [6], and *Lonicera japonica* [7].

Ferulic acid (FA, 4-hydroxy-3-methoxycinnamic acid), is a naturally occurring phenolic acid with many pharmacological activities. FA has been proven to be effective in many disease models such as depression, diabetes, and Alzheimer’s disease [8]. Moreover, FA is widely used in cosmetics due to its melanin blocking and tyrosinase inhibition activities [9]. To date, there was no comprehensive mechanism study of FA in anti-aging effects. Since FA acts as an antioxidant, we first focused on the antioxidant pathway involved in scavenging free radicals. The theory of Free Radical Aging states that the free radical and related oxidants cause damage to cellular constituents that ultimately cause structural and functional damage at tissue and organ levels [10]. Therefore, antioxidant supplementation is supposed to be an effective strategy to delay aging [11].

*C. elegans* is a suitable model organism for aging related experimentation because of its short lifespan, simple physiology, and genetic tractability [12]. Additionally, several fundamental genetic pathways that regulate aging process in *C. elegans* are conserved in mammals [12]. Here, we studied the lifespan-extending mechanism of FA in *C. elegans*.

## 2. Results

### 2.1. FA Extended C. elegans Lifespan

**Figure 1:**
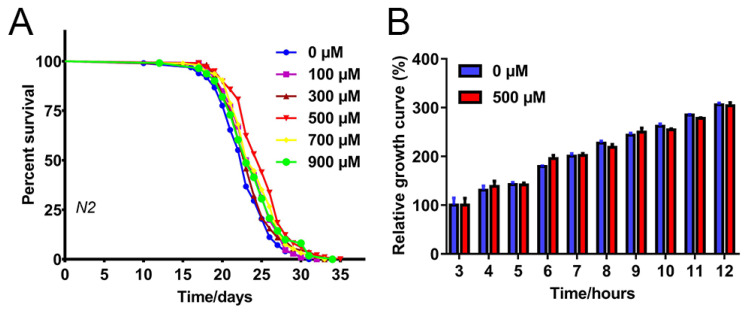
Effects of FA on lifespan in *C. elegans* and anti-bacterial in *E. coli* OP50. (**A**) FA extended *C. elegans* lifespan. Wild-type L4 larvae were treated with 0, 100, 300, 500, 700, 900 μM NGM at 20 °C. 500 μM FA treatment showed the most significant lifespan extension effect in *C. elegans*, n = 3 (~90 individuals per group), Kaplan–Meier survival analysis with Log-Rank test. (**B**) FA did not affect the growth rate of *E. coli* OP50, which was detected by OD600. Data were analyzed by Student’s *t*-test using GraphPad 7. Values were presented as mean ± SD.

To comprehensively explore the effect of FA on the lifespan of *C. elegans*, five different concentrations of FA, namely, 100, 300, 500, 700, and 900 μM, were tested at 20 °C. The mean lifespan of the nematodes with the treatment of 500 µM FA was significantly increased by 9.58% in comparison with the control group (Figure 1A, Table S3). This increase in lifespan was statistically significant when compared with the control group. The proliferation of bacteria can affect the lifespan of nematodes [13]. To explore whether the FA mediates lifespan extension by changing the properties of the bacterial food, we cultured *E. coli* OP50 (OP50) in Luria-Bertani (LB) medium with 500 μM FA. FA had no obvious inhibitory or growth-promoting effect on OP50 (compared to control, *p* > 0.05) (Figure 1B). Taken together, these results suggested that FA effectively extended the lifespan of *C. elegans* at 500 μM concentration. Therefore, the same concentration was used for subsequent experiments.

### 2.2. FA Partly Improved Healthspan in C. elegans

Typical markers of aging-relative phenotypes in *C. elegans* include the locomotory decline, pharyngeal pumping rate, gut granules, reproductive senescence, and morphological changes [14]. During the aging process, systemic muscle cells gradually lose vitality, which declines the mobility and pharyngeal pumping. Many studies have confirmed the antiaging effects on improving these conditions, such as tomatidine [15], protocatechuic acid [16], and carnosic acid [17].

**Figure 2:**
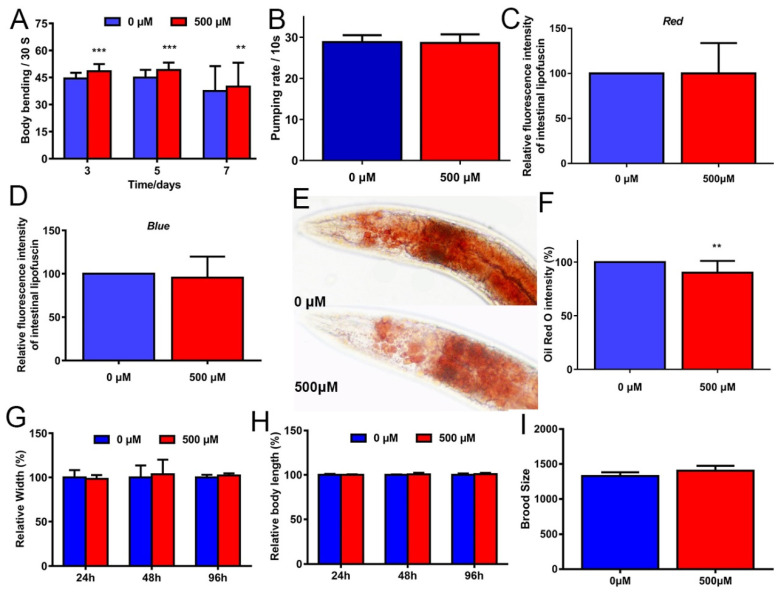
FA partly slowed age-associated physiological decline. (**A**) Effect of FA on body bending rate in N2 worms. (**B**) Effect of FA on pharyngeal pumping rate in N2 worms. (**C**,**D**) Effect of FA on intestinal lipofuscin in N2 worms. Two methods of identification were applied. (**E**,**F**) Effects of FA on fat accumulation. FA significantly decreased fat accumulation in N2 worms. (**G**,**H**) Effect of FA on body size in N2 worms. (**I**) FA did not affect reproduction in N2 worms. Data were analyzed by Student’s *t*-test using GraphPad 7. Values were presented as mean ± SD. *** *p* < 0.001, ** *p* < 0.01.

First, the locomotor performance of *C. elegans* can be expressed by swimming [18]. Therefore, we measured the body bends of N2 worms after exposure to the M9 buffer, which confirmed that FA may improve the locomotion of *C. elegans*. FA resulted in a significant improvement in the locomotor performance of worms after treatment on 3, 5, and 7 days (Figure 2A). Therefore, FA-treated for 5 days was selected as an optimal concentration for further experiments.

Second, intestinal lipofuscin accumulates with age, which can be used as a marker of health or the rate of aging. Two methods were used to detect intestinal autofluorescence [19]. FA seemly did not alleviate the accumulation of intestinal autofluorescence in N2 worms (Figure 2C,D).

Third, except for a progressive loss of muscle mass, aging is associated with an increase in visceral fat [20]. Exercise is an essential component of the treatment of obesity [21]. As explained previously, FA could effectively improve locomotor performance, without any significant difference in food intake. We hypothesized that the fat accumulation of *C. elegans* treated with FA would be reduced. To test this idea, N2 worms pretreated with or without FA were stained with Oil red O to investigate the effect of FA on fat accumulation. FA treatment decreased fat accumulation in the N2 worms (Figure 2E,F).

Last, an increased lifespan is generally accompanied by decreases in other physiological indices [6]. We compared the body length and body width of worms, either in the presence or absence of 500 µM FA. The result indicated that FA did not affect the body size of the worms (Figure 2G,H). We also studied the possible effect of FA on *C. elegans* fertility by counting the number of progenies. The result showed that FA had no obvious changes in the total number of offspring (Figure 2I), indicating the safety of FA.

In conclusion, FA partly alleviated aging-related declines in physiological performance in *C. elegans*.

### 2.3. FA Increased Stress Resistance and Reduced ROS Levels under Stress Conditions

**Figure 3:**
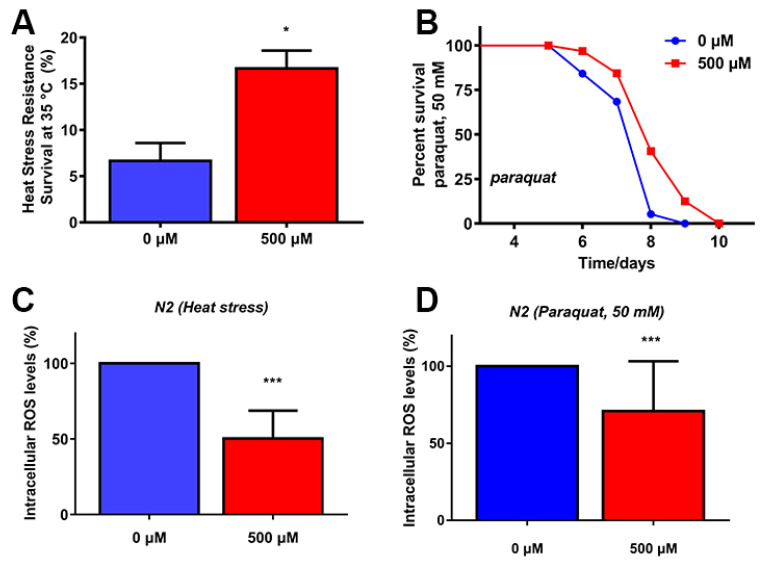
FA improved heat and oxidative stress resistance. (**A**) The average survival time of N2 worms cultured with 500 μM FA at 35 °C. (**B**) The lifespan of N2 worms cultured with 500 μM FA and 50 mM paraquat. (**C**,**D**) Effects of FA on ROS levels in N2 worms. FA effectively reduced ROS induced by heat stress and oxidative stress. Statistical analysis was performed by Student’s *t*-test or the log-rank test by GraphPad 7. Values were presented as mean ± SD, *** *p* < 0.001, * *p* < 0.05.

Increased lifespan is partly associated with improved survival under oxidative or heat stress. To investigate whether FA could enhance stress resistance, we pretreated L4 worms with 500 μM FA for 5 days, followed by exposure to paraquat (an intracellular ROS generator) or heat stress (35 °C). FA significantly increased the survival rate of *C. elegans* under heat stress or paraquat (Figure 3A,B). These results indicated that FA exerted protective roles against heat stress as well as oxidative stress in *C. elegans*.

Because both paraquat and heat stress cause mitochondrial damage by the accumulation of ROS [22,23], we next investigated the effect of FA on ROS levels under stress conditions using H_2_DCFDA, a fluorescent probe for ROS [24]. The result showed that FA treatment significantly suppressed the accumulation of ROS induced by heat stress as well as paraquat-induced compared to control group (Figure 3C,D). These results suggested that the increase in lifespan and stress resistance by FA treatment was due to free radicle scavenging activity of FA, which could be attributed to FA. Since FA is an effective antioxidant [25,26]. These results could give us some hint that FA-mediated mechanism could be predominantly attributed to the increase in stress resistance and lifespan which involved in oxidative stress response [7,27,28]. Therefore, we further investigated the mechanism of FA-mediated increase in stress resistance and lifespan in *C. elegans*, which may be the activation of pathways associated with oxidative stress responses and longevity.

### 2.4. FA-Mediated Lifespan Extension Did Not Depend on the Dietary Restriction

**Figure 4:**
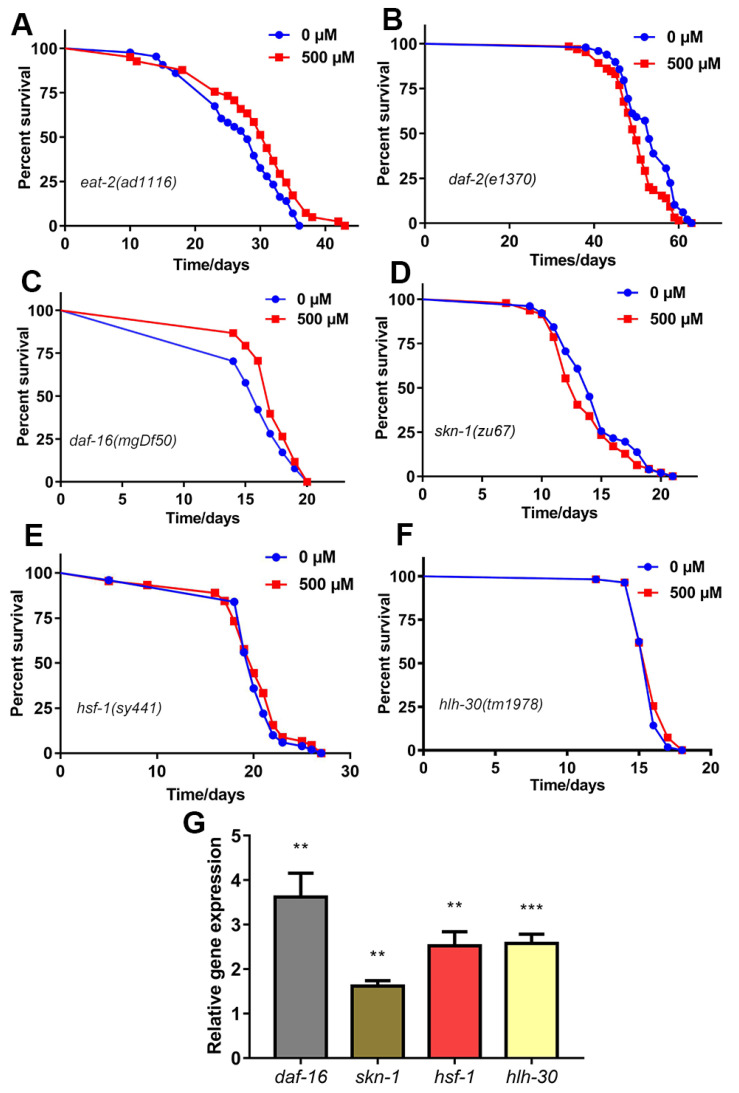
Mechanism of FA-mediated longevity. (**A**) FA extended lifespan in *eat-2(ad1116)* worms. 500 μM FA treatment significantly increased the lifespan of *eat-2(ad1116)* worms (**B**) Effect of FA-treated on lifespan in *daf-2(e1370)* worms. (**C**) Effect of FA-treated on lifespan in *daf-16(mgDf50)* worms. (**D**) Effect of FA-treated on lifespan in *skn-1(zu67)* worms. (**E**) Effect of FA-treated on lifespan of *hsf-1(sy441)* worms. (**F**) Effect of FA treatment on lifespan of *hlh-30(tm1978)* worms. Raised at 20 °C on NGM plates containing 500 μM FA or control group. (**G**) *daf-2*, *daf-16*, *skn-1*, *hsf-1*, and *hlh-30* mRNA levels in worms treated with 500 μM FA. Statistical analysis of the lifespan was performed using GraphPad 7 and *p* values were calculated by the log-rank test. Numerical data were analyzed by Student’s *t*-test and values were presented as mean ± SD, *** *p* < 0.001, ** *p* < 0.01.

Dietary restriction in yeast or rodents with a reduction in the food intake and without malnutrition, makes them live longer than organisms fed with a normal diet [29]. The effect of nutraceuticals may also reduce the food intake in animals to achieve the effect of dietary restriction [30,31]. To explore the effect of FA on dietary restriction on lifespan extension, we utilized a well-established dietary restriction genetic model, *eat-2(ad1116)*. The *eat-2(ad1116)* worms have a mutation in a nicotinic acetylcholine channel that reduces pharyngeal pumping and food intake, and thus extending the lifespan through restricted caloric intake [32]. FA treatment extended the lifespan in *eat-2(ad1116)* worms (Figure 4A, Table S3). Previous result showed that the pharyngeal pumping rate did not seem to be changed (Figure 2B). Therefore, these results indicated that FA-mediated effects on lifespan extension were independent of the dietary restriction pathway.

### 2.5. FA Extended C. elegans Lifespan through the Insulin Signaling Pathway

Insulin/IGF-1 signaling pathway(IIS) in the *C. elegans* is the central determinant of the endocrine control of stress response and aging [33]. The *daf-2* gene encodes the IGF-1 receptor. The *daf-2* mutant *C. elegans* exhibit a significant increase in lifespan [34]. DAF-16/FOXO is the major transcriptional output of IIS, which acts downstream of DAF-2/IIS in this pathway [35]. DAF-16/FOXO, a key transcription factor, modulates different signals to control aging, and improves lifespan via shuttling from cytoplasm to nucleus [36]. To understand the mechanism of FA for lifespan extension through the IIS pathway, we performed lifespan assay in *daf-2(e1370)* and *daf-16(mgDf50)* worms. We found that FA treatment did not extend the lifespans of *daf-2(e1370)* worms (Figure 4B, Table S3). However, FA treatment extended the lifespan of *daf-16 (mgDf50)* worms (Figure 4C, Table S3). It was surprising to note that qRT-PCR results showed that FA increased the mRNA level of *daf-16* in comparison with control group (Figure 4G). Therefore, we concluded that the longevity effect of FA required *daf-2*, and may be mediated partly by the transcription factor, DAF-16/FOXO. 

### 2.6. SKN-1 Was Required for FA-Mediated Lifespan Extension

SKN-1 is the *C. elegans* ortholog of mammalian Nrf/CNC proteins and is best known as a regulator of antioxidant and xenobiotic defense [37]. DAF-2/IIS inhibits directly DAF-16/FOXO and SKN-1/Nrf2 in parallel [38]. These studies raised the possibility that FA may mediate longevity through the parallel pathway SKN-1. We confirmed that FA could activate SKN-1 as observed from increased mRNA level of *skn-1* in N2 worms (Figure 4G). We also demonstrated the longevity effect of FA was completely lost in *skn-1 (zu67)* worms (Figure 4D, Table S3). Collectively, these results suggested that SKN-1 was required to prolong the lifespan of *C. elegans*.

### 2.7. HSF-1 Was Required for FA-Mediated Lifespan Extension

Highly conserved heat-shock transcription factor-1 (HSF-1) regulating by IIS is essential for improving lifespan and resistance stress [39,40]. HSF-1 regulates the expression of a set of proteins calling heat shock proteins (HSPs). To investigate the mechanism of FA-mediated lifespan extension through HSF homolog, *hsf-1*, we analyzed the effect of 500 μM FA on the mRNA level of *hsf-1* gene in N2 worms by qRT-PCR. The mRNA level of *hsf-1* were remarkably increased after FA treatment (Figure 4G). However, FA failed to extend the lifespan of *hsf-1 (sy441)* worms (Figure 4E, Table S3) suggesting that *hsf-1* was required for its lifespan extension.

### 2.8. HLH-30 Was Required for FA-Mediated Lifespan Extension

DAF-16/FOXO and HLH-30/TFEB can form a complex combinatorial transcription factor to promote stress resistance and lifespan in the same genetic pathway [41]. Recently research teams reported that FA can significantly activate the nuclear localization of HLH-30::GFP against amyloid-beta (Aβ) toxicity in *C. elegans* [42]. DAF-16 and HLH-30 are aging-regulatory transcription factors of significant importance and co-regulate several target genes in the promotion of lifespan [41]. Given that the longevity effect of FA may require DAF-16, we tested the role of HLH-30 in FA-mediated lifespan extension. FA failed to extend the lifespan of *hlh-30(tm1978)* worms (Figure 4F, Table S3). We further noticed remarkable increase in mRNA levels of *hlh-30* after FA treatment (Figure 4). These results suggested *hlh-30* was required for its lifespan extension.

### 2.9. FA Enhanced Expression of Anti-Oxidative Stress Genes and Autophagy Gene in C. elegans

**Figure 5:**
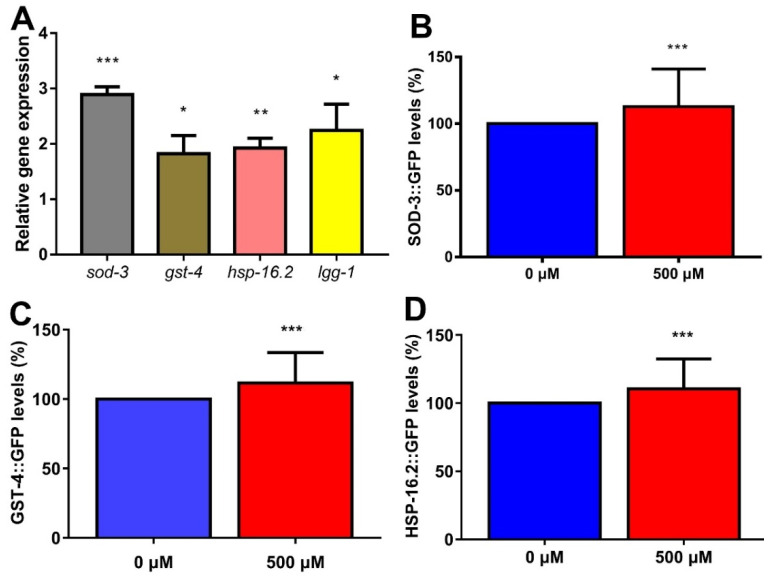
FA-mediated effects were related with antioxidant and autophagy genes. (**A**) FA significantly induced the mRNA expression of *sod-3*, *gst-4*, *hsp-16.2* and *lgg-1*. (**B**) FA significantly induced the expression of SOD-3::GFP. (**C**) FA significantly induced the expression of GST-4::GFP. (**D**) FA significantly induced the expression of HSP-16.2::GFP after incubation at 37 °C for 30 min. The images were analyzed with ImageJ software and numerical data were analyzed by Student’s *t*-test using GraphPad 7. Values were presented as mean ± SD, *** *p* < 0.001, ** *p* < 0.01, * *p* < 0.05.

FA has strong antioxidant effects in *C. elegans* and significantly scavenge the ROS generation under oxidative stress conditions. The targets of SKN-1/NRF-2 appear to be shared with DAF-16/FOXO [38]. Many cytoprotective and detoxification genes, such as superoxide dismutase (SOD-3) and glutathione S-transferase (GST-4) are regulated by the IIS-dependent transcription factor DAF-16 and SKN-1 [38]. qRT-PCR results showed that expression of *sod-3* and *gst-4* mRNA was significantly increased in worms exposed to 500 μM FA for 5 days (Figure 5A). Moreover, we measured the expression of GST-4 in a transgenic strain expressing GST-4::GFP. Simultaneously, the transgenic *C. elegans* SOD-3::GFP was employed to examine the effects of FA on levels of the antioxidant enzyme SOD-3. The result from the quantitative analysis showed significant upregulation of expression of SOD-3::GFP and GST-4::GFP (Figure 5B,C), suggesting that FA enhanced the expression of anti-oxidant stress genes.

The overexpression of cytoplasmic stress reporter *hsp-16.2* could extend lifespan [43]. The qRT-PCR analysis revealed a remarkable increase in mRNA level of *hsp-16.2* after FA treatment (Figure 5A). Moreover, there was 10.5% increase in relative mean fluorescence intensity of HSP-16.2::GFP (Figure 5D). HLH-30/TFEB is a master transcription factor that regulates many longevity pathways [44]. HLH-30 may function as a transcriptional regulator to mediate *lgg-1*-dependent autophagy [45]. The qRT-PCR result showed a remarkable increase in mRNA level of *lgg-1* after FA treatment (Figure 5A). 

All these results indicated the strong influence of FA on prolonging lifespan and improving stress resistance was dependent on the related stress-inducible genes as well as autophagy genes.

### 2.10. FA Decreased the ROS Levels in SKN-1 and DAF-16 Signaling Dependent Manner

**Figure 6:**
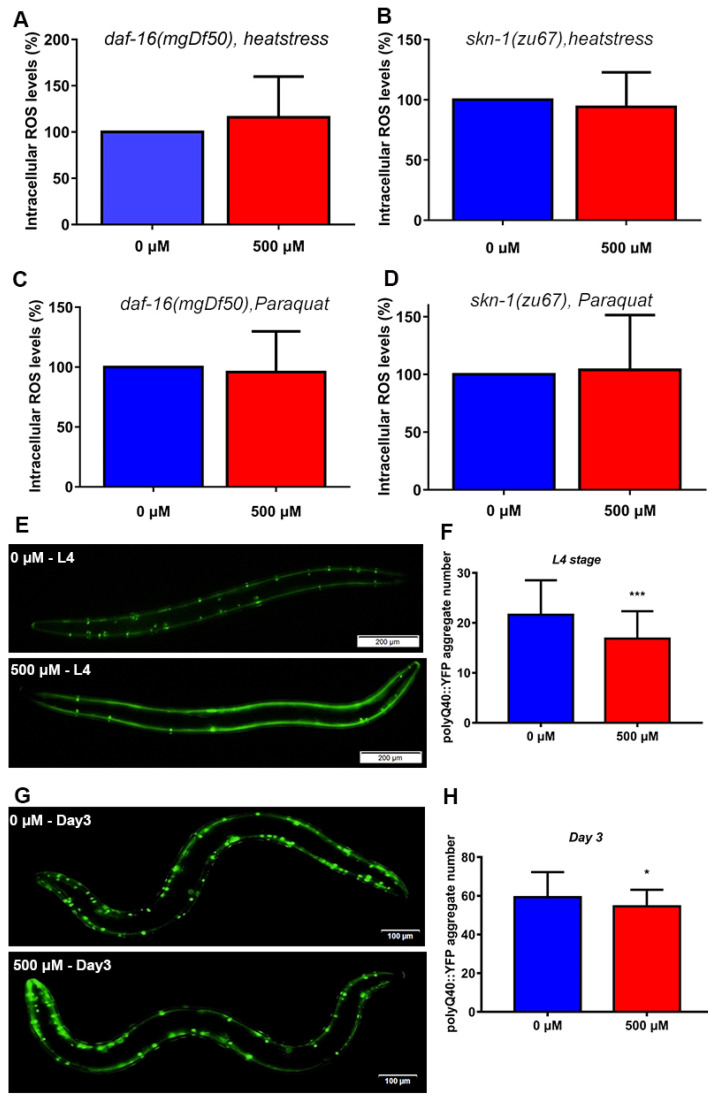
FA reduced ROS in *daf-16(mgDf50)* and *skn-1(zu67)* mutants. (**A**,**B**) FA reduced heat stress-induced ROS in *daf-16(mgDf50)* and *skn-1(zu67)* worms. (**C**,**D**) FA reduced paraquat-induced ROS in *daf-16(mgDf50)* and *skn-1(zu67)* mutants. (**E**–**H**) FA reduced protein aggregation regardless of administration at eggs and L4 stages. The number of aggregation spots of polyQ was counted. The ROS levels were analyzed with ImageJ software and numerical data were analyzed by Student’s *t*-test using GraphPad 7. Values were presented as mean ± SD, *** *p* < 0.001, * *p* < 0.05.

We investigated the ROS scavenging ability of FA under oxidative stress conditions in N2 worms. However, the exact pathway involved in FA scavenging ROS in nematodes was unknown. We treated *daf-16(mgDf50)* and *skn-1(zu67)* mutant animals with 500 μM of FA for 5 days at 20 °C, followed by exposure to 50 mM paraquat and 35 °C heat to induce oxidative and heat stress, respectively. Results shown that the FA-mediated reduction in ROS levels disappeared, suggesting FA reduced the ROS levels in SKN-1 and DAF-16 dependent manner (Figure 6A–D).

### 2.11. FA Reduced polyQ40 Aggregation in Transgenic C. elegans AM141

Defects in autophagy are critically associated with aging and aging-related diseases [46]. HLH-30 regulates autophagy and modulates longevity in *C. elegans* [47]. Moreover, HSF-1 and HSPs inhibit the onset of polyglutamine protein aggregation in animals [48]. Previous studies reported that HSP-16.2 suppresses the misfolded protein aggregation in *C. elegans* [43]. FA activated the mRNA expression of *hsf-1*, *hlh-30*, and its downstream targets (Figure 4G and Figure 5A). Therefore, we used a transgenic *C. elegans* AM141 strain as Huntington’s disease model and examined the effect of FA on protein aggregation in vivo, with special focus on polyglutamine peptide aggregation in muscle cells of the worms. FA treatment could significantly reduce the number of polyQ40::GFP (Figure 6E–H).

### 2.12. Genome-Wide Transcriptional Profiling of N2 C. elegans

**Figure 7:**
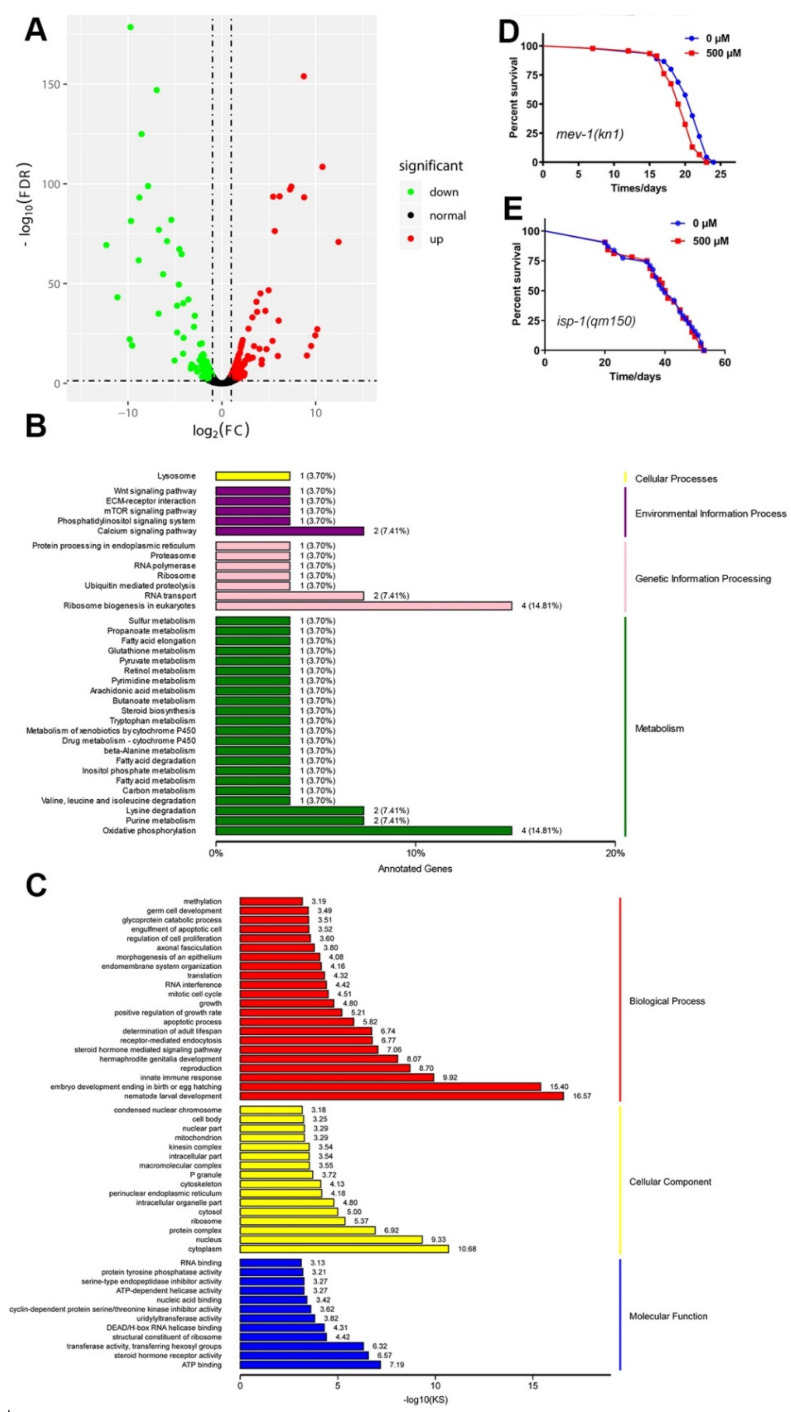
Genes were regulated by 500 μM FA and mitochondrial signaling pathway was necessary to prolong lifespan. (**A**) Volcano map showed the regulated genes that were significantly changed. There were 185 genes upregulated and 108 genes downregulated in FA-treated, compared to control group. (**B**) Top KEGG pathways enriched by the differentially expressed genes in N2 worm. (**C**) The GO analysis clarifies the molecular function, biological process, and cellular component of the differentially expressed genes in N2 worms. (**D**) Effect of FA on lifespan of *mev-1(kn1)* worms. (**E**) Effect of FA on lifespan of *isp-1(qm150)*. Worms raised at 20 °C on NGM plates containing 500 μM FA or control group.

We carried out RNA-seq to understand the mechanism of FA-mediated lifespan extension. Differentially expressed genes in 500 μM FA compared to controls visualized with a volcano plot (Figure 7A). 108 genes were differentially down-regulated, and 185 genes were up-regulated compared to control group (FDR < 0.01 and Fold Change ≥ 2). A larger number of GO terms were found enriched in ATP binding, cytoplasm, steroid hormone receptor activity, and nematode larval development (Figure 7C). These enrichments were related to growth control and energy consumption, suggesting a correlation between energy metabolism and longevity. Supplementation of FA enhanced the expression of genes in oxidative phosphorylation (Figure 7B). These results indicated the potential role of the mitochondrial pathway in FA treatment-related lifespan promotion.

The KEGG (Kyoto Encyclopedia of Genes and Genomes) mapper analysis showed a dramatic change in several physiological processes involved in metabolism, including fatty acid metabolism, purine metabolism, amino acid metabolism, carbon metabolism, butanoate metabolism, and arachidonic acid metabolism (Figure 7B). Pyruvate metabolism was up-regulated in FA-treated nematodes. Pyruvate is decarboxylated by pyruvate dehydrogenase complex to produce acetyl-coA, which is the key substance for ATP synthesis and synthesis of acetylcholine [49]. Table S4 showed that Monocarboxylate Transporter 1 (*mct-1*) was one of the most significant up-regulated gene. Previous studies have shown that the solute transporter gene *mct-1/2* overexpression is enough to extend the lifespan of N2 worms [50]. qRT-PCR results confirmed the FA upregulated *mct-1* mRNA, and showed that *mct-1* mRNA had no significant difference in *daf-2(e1370)* worms after FA-treated (Figure S1). Previous study suggested that FA was transported via a monocarboxylic acid transporter in Caco-2 cell [51]. We speculated that FA may be transported into the cells by MCT-1 in *C. elegans*, and further upregulated *mct-1* mRNA and multiple signal pathways to prolong the lifespan.

Comprehensively, all observations on the lifespan-extending effects of FA, due to altered metabolic homeostasis as a hallmark of lifespan regulation indicated that metabolism and aging are intimately linked [52,53]. Our transcriptome data suggested that FA regulated metabolic pathways to exert its antiaging effect in *C. elegans*.

### 2.13. A Mitochondrial Pathway Was Required for FA-Mediated Lifespan 

Next, our results showed an association between increased oxidative phosphorylation and positive life benefits. Moreover, mitochondria generate ROS that are thought to augment intracellular oxidative stress [54]. Paraquat induced oxidative stress resulted in mitochondrial dysfunction, which significantly affected the longevity and respiratory chain [55]. According to the mitochondrial free radical theory of aging, the accumulation of oxidative damage to macromolecules in the mitochondria is the causative mechanism for aging [56]. Given that FA could effectively reduce paraquat-induced ROS generation, we investigated mitochondrial function role in FA-mediated lifespan extension. The *isp-1* gene encodes mitochondrial complex III, and worms with the mutation of *isp-1* gene were long-lived [56]. As opposed to *isp-1*, the mutation of *mev-1*, encodes the orthologous form of succinate dehydrogenase cytochrome b560 subunit in complex II of the mitochondrial electron transport chain [57]. Our results showed that FA failed to extend the lifespan of the *isp-1(qm150)* worms (Figure 7E, Table S3) and *mev-1(kn1)* worms (Figure 7D, Table S3), and demonstrated that mitochondrial signaling was required for FA-mediated lifespan.

## 3. Discussion

FA is a phenolic compound derived from vegetables and fruits, is well-known as a potent antioxidant of scavenging free radicals. Many studies in humans and model organisms showed that FA had a variety of health benefits like antioxidants, anti-inflammatory, modulation of enzyme activities, activation of transcriptional factors, gene expression, and signal transduction in biological systems [8]. However, the mechanism by which FA slows aging was unknown. Our results showed that FA was an effective nutritional agent for extending lifespan in *C. elegans* (Figure 1A). 500 μM FA treatment was effective at extending lifespan. Moreover, it was unlikely that FA prolonged the lifespan of *C. elegans* by affecting bacterial food source (Figure 2B). The extension of lifespan must be accompanied by the change of physiological. Next, we investigated the effect of FA on the physiological of worms. FA resulted in a significant improvement in the locomotor performance (Figure 2A). FA did not change pharyngeal pumping rate. Moreover, FA treatment extended the lifespan in *eat-2* worms. These results indicated that dietary restriction may be not involved in FA-mediated lifespan extension (Figure 3A). ROS-mediated oxidative stress plays a pivotal role in the process of aging [16]. In this regard, we explored the effects of FA on the survival of worm under heat stress and oxidative stress. The result showed that FA significantly improved the survival of *C. elegans* in harsh environments (Figure 6A,B). Moreover, paraquat and heat shock cause mitochondrial damage by accumulation of ROS. We dissected the effect of FA on ROS in vivo using *C. elegans* model and found FA significantly reduced ROS levels when comparing to control group (Figure 3B,C). The above results proved that FA had excellent antioxidant capacity in vivo. We speculated that the lifespan extension of FA may activate pathways particularly associated with oxidative stress responses and longevity.

Insulin/IGF-1 signaling pathway is the central determinant of the endocrine control of stress response, diapause and aging. qRT-PCR analysis revealed that the RNA levels of genes involved in insulin/IGF-1 signaling pathway (Figure 4G). FA did not extended lifespan of *daf-2(e1370)* worms (Figure 4B), but extended lifespan of *daf-16(mgDf50)* worms which indicated DAF-16 was not necessary for FA to prolong life (Figure 4C). Previous study has shown that DAF-16/FOXO and HLH-30/TFEB can form a complex as combinatorial transcription factors to promote stress resistance and longevity in the same genetic pathway [41]. Indeed, HLH-30 was involved in FA-mediated lifespan extension. SKN-1 controls acute transcriptional response to oxidative stress and senses mitochondrial function and mitochondrially produced ROS [37,58]. We found that *skn-1* was involved in FA-mediated lifespan extension. Moreover, HSF-1 also acts as a key regulator of the rate of organismal aging and heat shock response [59]. FA did not extended lifespan of *hsf-1(sy441)* worms, which indicated *hsf-1* was required for FA-mediated lifespan extension. We further examined whether effects of FA on longevity and stress resistant were related to antioxidant- or autophagy-associated genes, examining transcription of *sod-3*, *gst-4*, *hsp-16.2,* and *lgg-1* [7]. We found that the mRNA of these genes was remarkably increased after FA treatment. Simultaneously, the effect of FA on antioxidant enzyme (SOD-3) and glutathione S-transferase (GST-4) were studied by the transgenic worms. We found that FA increased the levels of SOD-3::GFP and GST-4::GFP (Figure 5B,C). The targets of SKN-1 appear to be shared with DAF-16. It was reasonable to considered that *daf-16* and *skn-1* were involved in the resistance to external stress. The result of the experiment confirmed this hypothesis. The decrease of ROS levels by FA disappeared in the *daf-16(mgDf50)* and *skn-1(sy441)* worms (Figure 6A–D). HSP-16.2 is used to test stress levels in worms and regulated by HSF-1 [60]. FA increased the expression of HSP-16.2::GFP when comparing to control group. The expression of autophagy gene *lgg-1* decreased when knocked out *daf-16* and *hlh-30* [46]. FA increased the mRNA expression of *lgg-1* which indicated autophagy may involve in FA-mediated beneficial effect (Figure 5A). Autophagy is also the major cellular pathway involved in misfolded protein degradation [61]. Therefore, we examined the effect of FA on protein aggregation in transgenic *C. elegans*. FA could effectively reduce protein aggregation (Figure 6E–H).

To fully understand the mechanism of FA, Gene ontology analysis (GO, http://www.geneontology.org/, accessed on: 15 March 2020) of the differentially expressed mRNAs in response to FA treatment was shown in Figure 7C. The data were presented according to the following categories: Biological processes, molecular functions and cellular components. The most represented molecular functions identified by GO enrichment analysis were regulation of ATP binding. The mitochondrial respiratory chain plays a crucial role in energy metabolism, which was an important part of ATP production [62]. In the recent years, evidence has accumulated that life span is considerably influenced by the regulation of the complex interplay between cellular components like transcripts, proteins, and metabolites [63]. Manipulating amino acid intake in *Drosophila* and *C. elegans* can profoundly affect lifespan and reproduction [64]. 20 proteogenic amino acids except phenylalanine and aspartate extended lifespan [65]. Amino acid metabolites in *C. elegans* suggesting that anaplerosis of tricarboxylic acid (TCA) cycle substrates likely plays a role in lifespan extension [65]. Monocarboxylate transporters catalyze the proton-linked transport of monocarboxylates such as L-lactate, pyruvate, and the ketone bodies across the plasma membrane [66]. Changes in many metabolic pathways, we speculated that FA may regulate metabolic pathways to exert its antiaging effect in *C. elegans.* Animals should not simply live longer, but also display prolonged activity that resembles younger animals [67]. FA promoted healthspan, including locomotion in liquid, resistance to heat stress and oxidative stress. FA upregulated ECM-receptor interaction pathways (extracellular matrix). ECM remodeling is critical for longevity [68]. It seems that FA may regulate extracellular matrix remodeling to extended lifespan. Mitochondrial electron transport is a key determinant of lifespan in *C. elegans.* GO enrichment analysis and KEGG pathway were point to mitochondrial function. Two mutations related to mitochondrial electron transport chain (ETC) were used in lifespan assay. The result showed that mitochondrial pathway was required for FA-mediated lifespan extension. *isp-1(qm150)* worms have been shown to have a decreased rate of oxidative phosphorylation [69]. KEGG pathway analysis result showed that the genes of oxidative phosphorylation were significantly increasing. These results suggested it was quite necessarily for maintaining the normal oxidative phosphorylation of FA-mediated lifespan extension.

**Figure 8:**
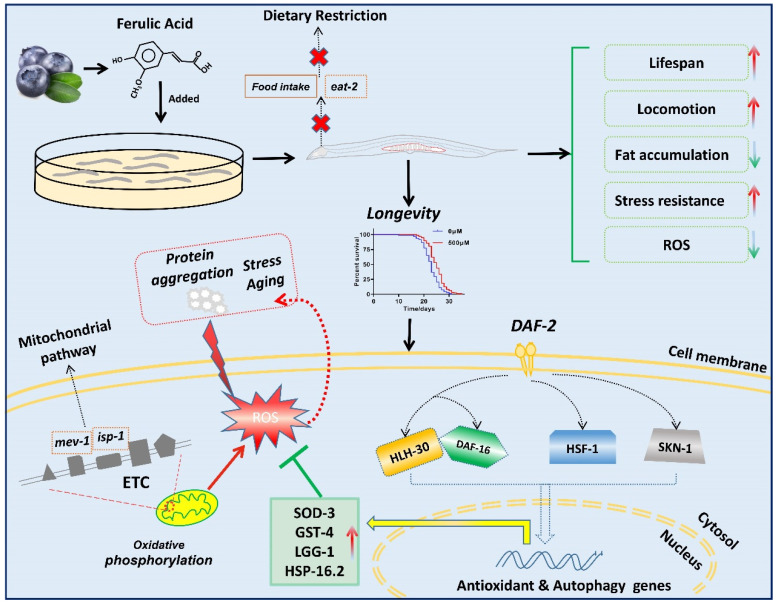
Schematic representation of mechanism of FA extended lifespan.

The Figure 8 showed that the mechanisms of FA increased lifespan and stress resistant in *C. elegans*.

## 4. Materials and Methods

### 4.1. Preparation of Ferulic Acid and E. coli OP50

Ferulic acid (FA, 98%, Aladdin, Shanghai, China) was prepared into 0.2% DMSO stock solution (*v/v*). FA powder was dissolved in dimethyl sulfoxide (DMSO) and diluted by water. The stock solution was sterilized by filtration through 0.2 μm pore size membranes. A 0.2% DMSO containing various concentrations of FA stock solution, portion 1:1, diluted by OP50 prior to administration. The mixture was pipetted onto nematode growth medium (NGM) plates 1 day before use. OP50 incubated with continuous shaking at 200 rpm at 37 °C for 12 h and concentrated threefold before use.

### 4.2. Lifespan Experiment

Lifespan assay were performed as described previously with some modifications [70]. Synchronized worms were cultured on OP50-seeded NGM plates at 20 °C or 16 °C (*daf-2(e1370)* worms) until reaching L4 stage and then transferred to new. The NGM plates containing 50 μM 5-fluoro-2′-deoxyuridine (FUdR, Aladdin, Shanghai, China) to prevent their progeny from hatching were seeded with a mixture of bacteria/FA. A previous study showed that 50 μM FUdR had no significant effect on the lifespan of N2 worms [71]. At least 80 hermaphrodite worms were examined per treatment and observed every day. The number of live and dead worms was counted daily until all individuals died. All the *C. elegans* strains were shown in Table S1.

### 4.3. Antibacterial Assay

Briefly, the FA stock solution was diluted in a 96-well microtiter plate with OP50. Aliquot of 100 μL of the bacterial suspension was incubated at 37 °C and read in an Infinity 200 Pro microplate reader (Tecan, Switzerland). OD600 value was recorded every hour.

### 4.4. Lipofuscin Assay

Briefly, worms were anesthetized on the 12th day after FA treatment. The images were captured using an Olympus X71 fluorescence microscope (Tokyo, Japan). Approximately 30 worms were measured and detected by the red fluorescence (Ex/Em 546/600 nm) and blue fluorescence (Ex/Em 350/460 nm). Scores were the average age pigment fluorescence intensity levels of three independent trials using ImageJ 15.2v software [72].

### 4.5. Body Bend Assay 

Locomotive ability was determined as described earlier [7]. To measure the frequency of body bending, 30 worms were treated with various concentrations of FA for 3, 5, or 7 days on NGM plates without food and with 10 μL of M9 buffer on the top of the agar. The number of sinusoidal curves made during locomotion in 30 s was scored [72].

### 4.6. Pharyngeal Pumping Assay 

For pharyngeal pumping experiments, worms (~20) were treated with or without 500 μM FA for 3, 5, or 7 days on NGM plates. The pharyngeal pump was recorded on a COIC stereoscope (BK1201, Chongqing, China) and was counted for 10 s.

### 4.7. Reproduction Assay 

Five synchronized L4 larvae were randomly transferred to fresh NGM plates treated with or without 500 μM FA. They were transferred onto a fresh NGM plate every 24 h. The eggs were then allowed to hatch and were counted at the L2 or L3 stage. The total number of progenies was referred as the initial reproduction.

### 4.8. Body Length and Body Width Assay 

Eggs were randomly transferred to fresh NGM plates treated with or without 500 μM FA. Recorded as 0 h, the growth of 24, 48, and 96 h were photographed, and the body length and body width were measured by ImageJ 15.2v. Body length was measured from the top of the head to the tip of the tail by using segmented line tools.

### 4.9. Oil Red O Staining

Oil Red O (Aladdin, Shanghai, China) staining was performed as described previously with some modifications [73]. To investigate fat accumulation after treatment with 500 μM FA for 5 days, approximately 30 worms were harvested by washing twice with M9 buffer. 1% Oil red O solution in isopropanol (Aladdin, Shanghai, China) was diluted in 2% Triton X-100 to prepare the working solution. The worms were fixed with a working solution for 20 min. The stained worms were washed with M9 buffer and subsequently placed on a 2% agarose gel pad. ImageJ 15.2v was used for the quantification of Oil red O mean intensity [72], and the background signals were subtracted.

### 4.10. Quantification of Reactive Oxygen Species (ROS) Production

ROS formation was quantified with 2′,7′-Dichlorofluorescein diacetate (H_2_DCFDA) (Meilunbio, China, Ex/Em 470/550 nm) [27]. Briefly, worms were maintained and treated as described above. After exposure to FA for 5 days, worms were washed off the plates with cold M9 buffer. OP50 were removed by repeated washes. Then, the nematodes were incubated with 100 μM H_2_DCFDA for 30 min at 35 °C or 50 mM paraquat for 6 h. The assay was repeated three times. ImageJ 15.2v was used to quantify the fluorescence intensity [72], and the background signals were subtracted. The polygon tool will be selected to depict the whole animal body and integrated density will be used to analyze the relative fluorescence intensity.

### 4.11. Thermorecovery Assay

Thermorecovery assays were determined as described [74]. NGM plates contain 30 animals per plate after FA treatment and animal survival was measured after 4 h at 35 °C followed by a recovery period of 12 h at 20 °C. A worm not responding to any mechanical stimuli was considered as dead.

### 4.12. Oxidative Stress Assay

Paraquat induced oxidative stress assay was conducted by incubating the worms for pretreatment with FA for 24 h [75]. Afterward, worms were transferred to fresh NGM plates containing 50 mM paraquat (Aladdin, Shanghai, China) at final concentration and FA. The survival of 30 worms was recorded every 24 h. A worm not responding to any mechanical stimuli was considered as dead. For statistical analysis, the log-rank test was used.

### 4.13. Fluorescence Imaging

Transgenic worms (CF1553, CL2166, TJ375) treated with or without 500 μM FA for 5 days, were anesthetized by using 10mM levamisole (Aladdin, Shanghai, China) and then placed on a 2% agarose pad. The expression of green fluorescent protein (GFP) was analyzed by imaging using a fluorescence microscope (Olympus X71, Tokyo, Japan). At least 60 worms were measured. For imaging, 470 nm was used for excitation and green fluorescence was recorded at 550 nm. ImageJ 15.2v was used to quantify the fluorescence intensity [72], and the background signals were subtracted. The polygon tool will be selected to depict the whole animal body and integrated density will be used to analyze the relative fluorescence intensity.

### 4.14. Analysis of PolyQ Strains

The number of intestinal PolyQ aggregates was counted in individual animals on day 3 or from egg to L4 of FA treatment. Animals were imaged on a 2% agarose pad after anesthesia with 10 mM levamisole. For images were determined as Fluorescence imaging. PolyQ40::YFP aggregates puncta were counted in each worm, at least 40 worms were measured. For imaging, 470 nm was used for excitation and green fluorescence was recorded at 550 nm. The result presented the number of punctate by observing with the naked eyes and counting.

### 4.15. Gene Expression Analysis by Quantitative Real-Time PCR

Briefly, worms were treated with or without 500 μM FA for 5 days as a lifespan assay. Total RNA was extracted from ~2000 worms per experiment using the Trizol reagent (TransGen Biotech, Beijing, China). The first-strand cDNA was prepared using the qRT-PCR kit (Bimake, Houston, TX, USA). qRT-PCR was performed using the Prism 7500 Real-Time PCR System (ABI, USA) with SYBR^®^ PCR kit (Bimake, Houston, TX, USA) along with 0.5 μM primers and 1 μL cDNA in a 20 μL reaction volume. Relative fold-changes in transcripts were calculated using the 2^−ΔΔCT^ method. Expression of the *act-1* genes was used as an endogenous control to normalize the amount of mRNA obtained from a target gene. Primers were listed in Table S2.

### 4.16. RNA Sequencing 

Gene expression in N2 worms treated with 500 μM FA or vehicle control, at days 5, was assessed by Biomarker Technology Company (Beijing, China). A total amount of 1 μg RNA per sample was used as input material for the RNA sample preparations. Sequencing libraries were generated using NEBNext UltraTM RNA Library Prep Kit for Illumina (NEB, Ipswich, MA, USA) following the manufacturer’s recommendations and index codes were added to attribute sequences to each sample.

Use the specified genome as a reference for the analysis, the download address was: https://www.ncbi.nlm.nih.gov/assembly/GCF_000002985.6 (WBcel235, accessed on: 19 October 2019).

Quantification of gene expression levels: Gene expression levels were estimated by fragments per kilobase of transcript per million fragments mapped. The formula was shown as follows:

$$\mathrm{FPKM}=\frac{cDNAFragments}{MappedFragments\left(Millions\right)*TranscriptLength\left(kb\right)}.$$

Differential expression analysis of two samples was performed using the edgeR. The FDR < 0.01 and Fold Change ≥ 2 was set as the threshold for significantly differential expression.

More detailed methods will be provided in the Supplementary File.

GEO number: GSE171836

### 4.17. Statistical Analysis

All experiments were carried out in triplicate. Statistical analysis of the lifespan was performed using GraphPad 7 Software and *p* values were calculated by the log-rank test. Numerical data were analyzed by Student’s *t*-test and values were presented as mean ± SD. Statistical differences were considered significant at *p* < 0.05 (* *p* < 0.05; ** *p* < 0.01; *** *p* < 0.001).

## 5. Conclusions

Our study confirmed a lifespan extension effect and a modulation of oxidative stress response of FA. Its mechanism of action involved insulin/IGF-1 signal transduction from worms to mammals, these pathways are well conservative. Our findings demonstrated plant derived FA as a safe antioxidant with a strong potential to protect against environmental stress and help to delay aging. Based the results of various experiments carried out in this study, we were representing the mechanisms of action of FA on *C. elegans* for improving the lifespan and stress resistance in a schematic way (Figure 8).

## Data Availability

Not applicable.

## Supplementary Materials

The following are available online at https://www.mdpi.com/article/10.3390/ijms22084279/s1, Table S1. All strains used in this study, Table S2. List of primers used for the quantitative real-time reverse transcription-polymerase chain reaction, Table S3. Effects of ferulic acid on the lifespan of N2 and mutant *C. elegans*. *p* value compared to the control (0 μM) group, Table S4. Top 10 genes upregulated by treatment with 500 μM FA, Figure S1. FA upregulated *mct-1* mRNA and showed that *mct-1* was regulated by DAF-2. Materials and methods (RNA-seq).
