# Supplementary material for: Ferulic Acid Supplementation Increases Lifespan and Stress Resistance via Insulin/IGF-1 Signaling Pathway in C. elegans
Source: Int J Mol Sci. 2021 Apr 20;22(8):4279. doi: 10.3390/ijms22084279 (PMC8074393; doi:10.3390/ijms22084279)
Supplement: Supplementary file 1 [file ijms-22-04279-s001.zip › ijms-1167075-supplementary.pdf]

Table S1. All strains used in this study.

Strains	Genotype
N2	*C. elegans* wild isolate.
DA1116	eat-2(ad1116) II.
CB1370	daf-2(e1370) III.
GR1307	daf-16(mgDf50) I.
EU1	skn-1(zu67) IV/nT1 [unc-?(n754) let-?] (IV;V)
PS3551	hsf-1(sy441) I.
JIN1375	hlh-30(tm1978) IV.
TJ375	gpIs1 [hsp-16.2p::GFP].
CF1553	muIs84 [(pAD76) sod-3p::GFP + rol-6(su1006)].
CL2166	dvIs19 [(pAF15)gst-4p::GFP::NLS] III.
MQ887	isp-1(qm150) IV.

Table S2. List of primers used for the quantitative real-time reverse transcription-polymerase chain reaction

**Gene name**	**Primer sequence (5'to3')**
act-1F	CTACGAACTTCCTGACGGACAAG
act-1R	CCGGCGGACTCCATACC
sod-3F	CTCCAAGCACACTCTCCCAG
sod-3R	TCCCTTTCGAAACAGCCTCG
gst-4F	TGGCAAGAATGATGCCCTGT
gst-4R	GCCCTCAACTTGTCCTCCTC
hsf-1F	TTTGCATTTTCTCGTCTCTGTC
hsf-1R	TCTATTTCCAGCACACCTCGT
hsp-16.2F	GGTGCAGTTGCTTCGAATCTT
hsp-16.2R	TCTTCCTTGAACCGCTTCTTTC
lgg-1F	ACCCAGACCGTATTCCAGTG
lgg-1R	ACGAAGTTGGATGCGTTTTC
hlh-30F	CTCATCGGCCGGCGCTCATC
hlh-30R	AGAACGCGATGCGTGGTGGG
daf-16F	CTAACTTCAAGCCAATGCCACTA
daf-16R	TCCAGCTTGACTCAGCTCATGTC
mct-1F	CGAGGAAGTCGGGAAAGTCA
mct-1R	TCTGCCCAGATCGGTACAAG

Table S3. Effects of ferulic acid on the lifespan of N2 and mutant *C. elegans*. *p* value compared to the control (0 μM) group

Genotype	Treatment	Mean	±SD	%	*p* value (log-rank significance)
N2	0 μM	22.74	3.365		
	100μM	23.82	3.051	4.75	0.0437
	300μM	21.93	3.246	-3.58	0.1233
	500μM	24.92	3.379	9.58	<0.0001
	700μM	24.12	3.35	6.06	0.0058
	900μM	23.81	3.828	4.46	0.0181
eat-2(ad1116)	0 μM	26.88	6.59		
	500μM	29.15	7.83	8.44	0.027
daf-2(e1370)	0 μM	51.45	7.18		
	500μM	48.78	6.88	-5.19	0.0598
daf-16(mgDf50)	0 μM	16.23	1.99		
	500μM	17.15	1.83	5.67	0.0252
skn-1(zu67)	0 μM	14.35	2.93		
	500μM	13.55	2.96	-5.57	0.226
hsf-1(sy441)	0 μM	19.74	3.6		
	500μM	19.62	4.43	-0.6	0.5118
hlh-30(tm1978)	0 μM	15.71	0.91		
	500μM	15.87	1.09	1.02	0.2732
mev-1(kn1)	0 μM	20.24	3.083		
	500μM	19.07	2.863	-5.78	0.004
isp-1(qm150)	0 μM	38.67	0.6152		
	500μM	37.43	0.6081	-3.21	0.1809

Table S4. Top 10 genes upregulated by treatment with 500 μM FA

Gene name	Description #	Overview *
mct-1	MFS domain-containing protein	Is predicted to have transmembrane transporter activity.
F49C12.10	Methyltransf_11 domain-containing protein	Is predicted to have methyltransferase activity.
Y80D3A.8	hypothetical protein	Is predicted to have protein tyrosine phosphatase activity.
fbxc-4	F-box C protein	Is enriched in intestine and neurons.<br>Is affected by several genes including daf-16; daf-2; and eat-2.
eif-3.J	Eukaryotic Initiation Factor	Is predicted to have translation initiation factor activity.
ech-6	Probable enoyl-CoA hydratase, mitochondrial	Is an ortholog of human ECHS1 (enoyl-CoA hydratase, short chain 1).<br>Is predicted to have enoyl-CoA hydratase activity. Localizes to the mitochondrion.
ZC395.10	abnormal DAuer Formation	Is predicted to have Hsp90 protein binding activity and chaperone binding activity.
map-2	Methionine aminopeptidase 2	Is an ortholog of human METAP2 (methionyl aminopeptidase
T26C12.2	hypothetical protein	Is affected by several genes including daf-2; age-1; and clk-1.<br>Is affected by four chemicals including Tunicamycin; multi-walled carbon nanotube and cadmium.
F31E3.6	hypothetical protein	Is affected by several genes including daf-16; glp-1; and daf-12;<br>Is affected by four chemicals including Mianserin; metformin; and Sirolimus.

Data from NCBI (National Center for Biotechnology Information)

* Data from WormBase (https://wormbase.org/, accessed on, accessed on: 3, 15, 2020)

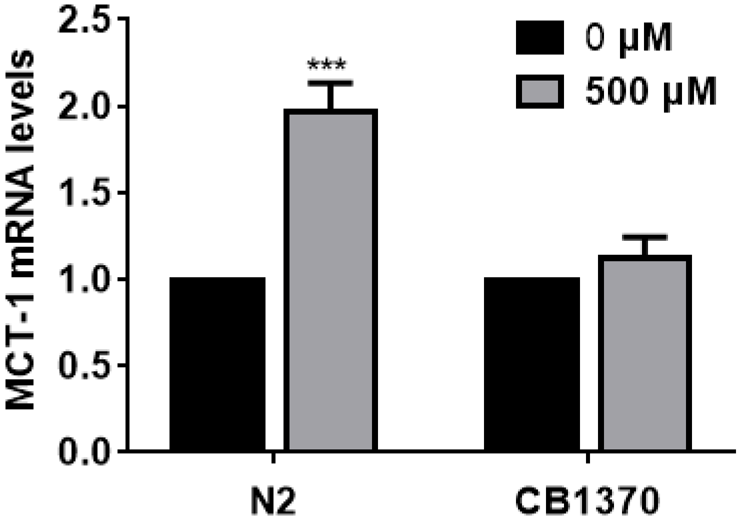

Figure S1. FA upregulated mct-1 mRNA and showed that *mct-1* was regulated by DAF-2.

Materials and methods (RNA-seq)

Materials and methods (RNA-seq)

1 Sample collection and preparation

1.1 RNA quantification and qualification

The purity, concentration and integrity of RNA samples are tested using advanced molecular biology equipment to ensure the use of qualified samples for transcriptome sequencing.

Library preparation for Transcriptome sequencing

A total amount of 1 μg RNA per sample was used as input material for the RNA sample preparations. Sequencing libraries were generated using NEBNext UltraTM RNA Library Prep Kit for Illumina (NEB, USA) following manufacturer's recommendations and index codes were added to attribute sequences to each sample. Briefly, mRNA was purified from total RNA using poly-T oligo-attached magnetic beads. Fragmentation was carried out using divalent cations under elevated temperature in NEBNext First Strand Synthesis Reaction Buffer（5X）. First strand cDNA was synthesized using random hexamer primer and M-MuLV Reverse Transcriptase（RNase H-）. Second strand cDNA synthesis was subsequently performed using DNA Polymerase I and RNase H. Remaining overhangs were converted into blunt ends via exonuclease/polymerase activities. After adenylation of 3' ends of DNA fragments, NEBNext Adaptor with hairpin loop structure were ligated to prepare for hybridization.

In order to select cDNA fragments of preferentially 200-250 bp in length, the library fragments were purified with AMPure XP system (Beckman Coulter, Beverly, USA). Then 3 μl USER Enzyme (NEB, USA) was used with size-selected, adaptor-ligated cDNA at 37°C for 15 min followed by 5 min at 95°C before PCR. Then PCR was performed with Phusion High-Fidelity DNA polymerase, Universal PCR primers and Index (X) Primer. At last, PCR products were purified (AMPure XP system) and library quality was assessed on the Agilent Bioanalyzer 2100 system.

1.2 Clustering and sequencing

The clustering of the index-coded samples was performed on a cBot Cluster Generation System using TruSeq PE Cluster Kit v4-cBot-HS (Illumia) according to the manufacturer's instructions. After cluster generation, the library preparations were sequenced on an Illumina Hiseq 2500 platform and paired-end reads were generated.

2 Data analysis

2.1 Quality control

Raw data (raw reads) of fastq format were firstly processed through in-house perl scripts. In this step, clean data (clean reads) were obtained by removing reads containing adapter, reads containing ploy-N and low-quality reads from raw data. At the same time, Q20, Q30, GC-content and sequence duplication level of the clean data were calculated. All the downstream analyses were based on clean data with high quality.

2.2 Comparative analysis

The adaptor sequences and low-quality sequence reads were removed from the data sets. Raw sequences were transformed into clean reads after data processing. These clean reads were then mapped to the reference genome sequence. Only reads with a perfect match or one mismatch were further analyzed and annotated based on the reference genome. Tophat2 tools soft were used to map with reference genome.

2.3 Gene functional annotation

Gene function was annotated based on the following databases: Nr (NCBI non-redundant protein sequences)； Nt (NCBI non-redundant nucleotide sequences)； Pfam (Protein family)；KOG/COG (Clusters of Orthologous Groups of proteins)；Swiss-Prot (A manually annotated and reviewed protein sequence database)；KO (KEGG Ortholog database)； GO (Gene Ontology).

2.4 SNP calling

Picard - tools v1.41 and samtools v0.1.18 were used to sort, remove duplicated reads and merge the bam alignment results of each sample. GATK2 software was used to perform SNP calling. Raw vcffiles were filtered with GATK standard filter method and other parameters (cluster WindowSize: 10; MQ0 >= 4 and (MQ0/(1.0*DP)) > 0.1; QUAL < 10; QUAL < 30.0 or QD < 5.0 or HRun > 5), and only SNPs with distance > 5 were retained.

2.5 Quantification of gene expression levels

Quantification of gene expression levelsGene expression levels were estimated by fragments per kilobase of transcript per million fragments mapped. The formula is shown as follow:

$$FPKM = \frac{cDNAFragments}{MappedFragments(Millions) * TranscriptLength(kb)}$$

2.6 Differential expression analysis

Differential expression analysis of two samples was performed using the edgeR. The FDR < 0.01 & Fold Change ≥2 was set as the threshold for significantly differential expression2.7 GO enrichment analysis

Gene Ontology (GO) enrichment analysis of the differentially expressed genes (DEGs) was implemented by the GOseq R packages based Wallenius non-central hyper-geometric distribution (Young et al, 2010), which can adjust for gene length bias in DEGs.

2.8 KEGG pathway enrichment analysis

KEGG (Kanehisa et al., 2008) is a database resource for understanding high-level functions and utilities of the biological system, such as the cell, the organism and the ecosystem, from molecular-level information, especially large-scale molecular datasets generated by genome sequencing and other high-throughput experimental technologies (http://www.genome.jp/kegg/). We used KOBAS (Mao et al., 2005) software to test the statistical enrichment of differential expression genes in KEGG pathways.

2.9 PPI (Protein Protein Interaction)

The sequences of the DEGs was blast (blastx) to the genome of a related species (the protein protein interaction of which exists in the STRING database: http://string-db.org/) to get the predicted PPI of these DEGs. Then the PPI of these DEGs were visualized in Cytoscape (Shannon et al, 2003).

References

Altschul S F, Madden T L, Schaffer A A, et al. (1997). Gapped BLAST and PSI-BLAST: a new generation of protein database search programs. Nucleic Acids Res. 25:3389-3402. (BLAST)

Anders S, Huber W. (2010). Differential expression analysis for sequence count data. Genome Biology, doi:10.1186/gb-2010-11-10-r106. (DESeq)

Finn R D, Tate J, Mistry J, et al. (2008). The Pfam protein family's database. Nucleic Acids Res 36, D281-D288. (Pfam)

Gotz S, Garcia-Gomez J M, Terol J, et al. (2008). High-throughput functional annotation and data mining with the Blast2GO suite.Nucleic Acids Research 36, 3420-3435. (BLAST2go)

Mao X, Cai T, Olyarchuk J G, et al. (2005). Automated genome annotation and pathway identification using the KEGG Orthology (KO) as a controlled vocabulary.Bioinformatics 21, 3787-3793. (KOBAS)

McKenna A, Hanna M, Banks E, et al. (2010). The Genome Analysis Toolkit: A MapReduce framework for analyzing next-generation DNA sequencing data. Genome Res. 20, 1297-1303. (GATK)

Kanehisa M, Araki M, Goto S, et al. (2008). KEGG for linking genomes to life and the environment. Nucleic Acids research 36: D480-D484. (KEGG)

Wang L, Feng Z, Wang X, et al. (2010) DEGseq: An R package for identifying differentially expressed genes from RNA-seq data. Bioinformatics 26, 136-138. (DEGseq)

Robinson M D, McCarthy D J, Smyth G K. (2010). edgeR: a Bioconductor package for differential expression analysis of digital gene expression data. Bioinformatics 26:139-140. (edgeR)

Li B, Dewey C. (2011). RSEM: accurate transcript quantification from RNA-Seq data with or without a reference genome. BMC Bioinformatics, doi:10.1186/1471-2105-12-323. (RSEM)

Young M D, Wakefield M J, Smyth G K, et al. (2010). Gene ontology analysis for RNA-seq: accounting for selection bias. Genome Biology, doi:10.1186/gb-2010-11-2-r14. (GOseq)

Shannon P, Markiel A, Ozier O, et al. (2003). Cytoscape: a software environment for integrated models of biomolecular interaction networks. Genome Res. 13, 2498-2504. (Cytoscape)
